# Investigation of the physicochemical, antioxidant, rheological, and sensory properties of ricotta cheese enriched with free and nano‐encapsulated broccoli sprout extract

**DOI:** 10.1002/fsn3.3001

**Published:** 2022-08-03

**Authors:** Zahra Azarashkan, Ali Motamedzadegan, Azade Ghorbani‐HasanSaraei, Pourya Biparva, Somayeh Rahaiee

**Affiliations:** ^1^ Department of Food Science and Technology, Ayatollah Amoli Branch Islamic Azad University Amol Iran; ^2^ Department of Food Science and Technology Sari Agricultural Sciences and Natural Resource University Sari Iran; ^3^ Department of Basic Sciences Sari University of Agricultural Sciences and Natural Resources Sari Iran; ^4^ Department of Microbial Biotechnology, Faculty of Biotechnology Amol University of Special Modern Technologies Amol Iran

**Keywords:** antioxidant activity, broccoli sprout, encapsulation, ricotta cheese, sensory evaluation, textural properties

## Abstract

This study aimed to produce the functional ricotta cheese using broccoli sprouts extract (BSE) and to evaluate its physicochemical, antioxidant, rheological, and sensory properties. The BSE nano‐liposome was nano‐encapsulated into basil seed gum (BSG) and was incorporated into the ricotta cheese formulation in two forms of free and nano‐capsules in two levels of 3% and 5% w/w. The measurements were conducted during a 15‐day storage period at 4–6°C. The results showed that the titratable acidity, hardness, and chewiness of cheeses were increased and the pH, moisture, total phenol content (TPC), and antioxidant activity were decreased (*p* < .05). With the addition of BSE concentration, the TPC and antioxidant activity increased significantly (*p* < .05) and applying the nano‐encapsulation method for BSE led to better preservation of bioactive compounds. Based on the rheological results, viscoelastic solid behavior and a weak gel were observed in all cheese samples. The results of sensory evaluation demonstrated that cheeses containing free extract had lower flavor and overall acceptability scores than other samples, which indicates that the nano‐encapsulation covered the undesirable flavor of the BSE. Generally, during the 15‐day cold storage period, the highest sensory acceptance and functional activity were related to the samples containing nano‐encapsulated BSE, especially at the 5% level.

## Graphical abstract

We were produced the functional Ricotta cheese using encapsulated broccoli sprouts extract (BSE) (3% and 5% w/w) nanoliposomes into basil seed gum successfully. Physicochemical, antioxidant, rheological, and sensory properties indicated that during a 15‐day storage period at 4‐6 C, the level of BSE 5% was the best. Viscoelastic solid behavior and a weak gel were observed in all cheese samples and the sensory evaluation indicated the nano‐encapsulation covered the undesirable flavor.

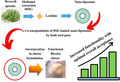

## 1 INTRODUCTION

Cheeses are one of the most popular dairy products consumed around the world that have a high commercial value (Christaki et al., 2021). Ricotta cheese is a soft cheese with high moisture content and is made from milk or cheese whey, or a mixture of both. Fresh ricotta cheese has a nutty and mild flavor. Ricotta cheese production is an economical way to use cheese whey (El‐Den, 2020). This type of cheese is traditionally prepared by heating whey and acidifying the hot whey liquid with acetic acid, citric acid, or lactic acid (to a pH of ~5) to coagulate the proteins of whey (Hesarinejad et al., 2021). No starter culture is required to manufacture ricotta cheese, and this cheese has a short shelf life even at a refrigerated temperature (Ricciardi et al., 2019). Ricotta cheese has a pleasant flavor and nutritional benefits, so it is widely used all over the world. Like other dairy products, this type of cheese has the ability to become a functional food product with improved health effects. Therefore, various natural additives, such as dietary fiber, natural antioxidants, vitamins, etc., can be used to convert ricotta cheese into a functional product (Siyar et al., 2022).

Today, the use of functional bioactive compounds in food products has attracted a lot of attention because functional products (Abedinia et al., 2021; Azarashkan, Farahani, et al., 2022), in addition to providing antioxidants, also have health benefits for consumers (Ali et al., 2021). Bioactive compounds and natural antioxidant agents derived from various sources play noticeable roles in body function maintenance, such as protecting the human body against various serious diseases, such as cardiovascular diseases, cancer, inflammations, diabetes, aging, blood pressure, and so on (Feng et al., 2019). Recent scientific research has shown that plants can be used as the main source of functional compounds in the food industry (Selahvarzi, Sanjabi, et al., 2021), and the development of functional dairy products with plants and herbs has been reported (Akan et al., 2021; El‐Sayed, 2020; Naji‐Tabasi & Razavi, 2017; Nzekoue et al., 2021). The herbs and their extracts are often rich and significant sources of bioactive materials, like vitamins, antioxidants, minerals, and phytochemicals such as essential oils, flavonoids, phenols, glycosides, alkaloids, tannins, organic acids, coumarin, and saponins (Motevalizadeh et al., 2018; Selahvarzi, Ramezan, et al., 2021).

Broccoli or *Brassica oleracea* L. var. *Italica*, Brassicacae is a main member of *Brassica* vegetables and is a rich source of vitamins (K, C, β‐carotene), fatty acids, polyphenols, and dietary fiber, polyphenols, and fatty acids, and has remarkable health beneficial effects (Petkowicz & Williams, 2020). One of the important parts of broccoli to obtain bioactive compounds is its sprouts (Paśko et al., 2018). Broccoli sprouts are a rich source of natural antioxidant compounds such as vitamins, carotenoids, minerals, sulfur compounds, and glucoraphanin (especially sulforaphane) (Kikuchi et al., 2021). Sulforaphane is an important bioactive compound found in different parts of broccoli that exhibits considerable and significant anti‐inflammatory and antioxidant activities (Yusin et al., 2021). In previous studies, the antioxidant activity and health effects of broccoli and sulforaphane as its bioactive compound have been approved (Cedrowski et al., 2021; González et al., 2021; Tian et al., 2017).

Many bioactive compounds are unstable and sensitive to changes in environmental conditions like light, pH, and temperature, and also show a residual flavor, so their use may therefore be limited. A significant solution to overcome these problems is to use the micro‐ or nano‐encapsulation process of functional and bioactive compounds (de Souza et al., 2020). The encapsulation process is a popular processing technology that increases bioavailability and improves the stability of bioactive agents. The encapsulated compounds provide remarkable health benefits. Therefore, they can be used as functional agents in food products (Yu et al., 2021). In encapsulation, various materials such as lipids, proteins, or carbohydrates are used to coat the bioactive compounds and agents (Esmaeilzadeh Kenari & Razavi, 2022; Ghobadi et al., 2021; Gómez‐Estaca et al., 2015). Recently, the functional activity of encapsulated bioactive compounds and herbal extracts in different dairy products, especially cheeses and yogurts, has been reported (Farrag et al., 2020; Flores‐Mancha et al., 2021; Matseychik et al., 2019; Motevalizadeh et al., 2018; Pérez‐Soto et al., 2021).

Various materials have recently been introduced as suitable wall materials for preserving bioactive compounds (Abedinia et al., 2017, 2018; Abedinia et al., 2020; Azarashkan, Farahani, et al., 2022). Basil seed gum (BSG) is a new plant hydrocolloid derivative of high molecular weight (2320 kDa) with intrinsic viscosity (39.17 dl gr^−1^) which is known as glucomannan anion heteropolysaccharide and consists of two main parts with different molecular weights (Mohammad Kheshtchin et al., 2022). Basil seed gum (BSG) is biodegradable and has hydrophilic nature, has low production cost, is heat‐resistant, and also shows desirable and acceptable rheological properties (Hashemi & Khaneghah, 2017). It exhibits emulsifying, thickening, binding, foaming, stabilizing, and gelling properties and therefore has many applications in the pharmaceutical and food industries (Kim et al., 2020). Research has approved that BSG, due to its special applications in coating and its osmotic effects in drying the coated core, can be used successfully for the encapsulation of active ingredients (Etemadi et al., 2020).

As mentioned earlier, broccoli sprouts contain a wide range of bioactive compounds and indicate different health benefits, so in this research, the production of functional ricotta cheese using the free and encapsulated broccoli sprout extract in BSE was investigated and the physicochemical, antioxidant, rheological, and sensory properties of the ricotta cheeses during refrigerated storage were studied.

## 2 MATERIALS AND METHODS

### 2.1 Materials

Broccoli seed and basil seed gum powder and lecithin with 99% purity were purchased from a local market (Sari, Iran), Park Laboratory of Science and Technology, and Across Co. (America), respectively. All chemicals and reagents used in this study were purchased from Sigma‐Aldrich Co.

### 2.2 Preparation of the broccoli sprouts extract

The broccoli seeds were soaked in water and placed in the dark for 8 h. The seeds were then poured on the sieve and placed in the dark for 7 days. The broccoli seeds were washed with purified water every 12 h. For the preparation of the BSE, the broccoli sprouts were dried in an oven at 40°C (Memmert, Germany) and pulverized. The broccoli sprout powder (0.1 g) was then added to 80% v/v methanol (20 ml) and stirred for 24 h. After that, the filtration was performed using Whatman paper No. 1. The solvent was then evaporated by a rotatory evaporator (Heidolph, Germany) at a temperature of 45°C.

### 2.3 Preparation of the nanocapsules

To prepare the BSE nano‐liposome according to the thin‐film hydration method (Pinilla & Brandelli, 2016), first, a certain amount of BSE (to reach the final level of 1.0% w/w DM (dry matter)) was dissolved in a chloroform/lecithin mixture. The solvent of the mixture was then evaporated using a rotatory evaporator (at 35°C for 10 min). Then, the micro‐liposome film was obtained after drying the concentrated solution in an oven under a vacuum. Then, the formed film was dissolved in 20 ml of a phosphate buffer solution with pH = 7 and was sonicated with 2 cycles of 2 min and 10 s of rest between each cycle at 400 Watt by a probe sonicator, and a monolayer nano‐liposome was produced. In the previous two studies (Azarashkan, Farahani, et al., 2022; Azarashkan, Motamedzadegan, et al., 2022), we considered different levels of gum and extract as variables and during those studies, we obtained the best treatments that we used in this study. BSE‐loaded nano‐liposome was then encapsulated with basil seed gum (BSG) (0.5% w/w). For this reason, BSG (to reach the final level of 0.5% w/w DM) was dissolved in water and stirred (at 1500 rpm). The BSG dispersion was then placed at refrigerated temperature for 24 h. After preparation of BSG solution, this solution was incorporated dropwise to the BSE‐loaded nano‐liposome suspension and stirred (at 800 rpm for 2 h). The BSE‐loaded nano‐capsule (L1BSG0.5) was finally dried by a freezing dryer (Operon FDB‐550, South Korea) for 19 h at a temperature of −70°C and then pulverized.

### 2.4 Preparation of ricotta cheeses

To prepare ricotta cheese, whole milk powder was dissolved in water and the pH of the solution was adjusted to 7 with NaOH, and then heated at 90°C for 30 min in a hot water bath. After that, citric acid (25 ml/L) was added. The cheese curd was formed and the cheese was collected in a mold (43 × 20 mm) to separate the whey. The cheese was cooled at room temperature (25 ± 2°C), and then its weight was recorded (45 g). The free and nano‐encapsulated BSE at two levels (3 and 5% w/w) were incorporated into the ricotta cheese and mixed by a hand mixer for 5 min. The cheese sample without BSE was considered as the control sample. The cheese samples were refrigerated (4–6°C) for 15 days and tested on the 1st, 7th, and 15th days.

### 2.5 Physicochemical parameters’ analysis

The moisture, fat, and protein contents of cheeses were measured according to the methods provided by Association of Official Analytical Chemists (AOAC) (2000). The moisture content of cheeses (% w/w) was determined by drying the samples (5 g) at 105 ± 3 °C to a constant weight. The protein and fat content (% w/w) was measured using micro‐Kjeldahl and Gerber methods, respectively. The pH of samples was measured using a digital pH meter. Titratable acidity values (% lactic acid) were determined by the titration of sample solution with 0.1 NaOH (Manzo et al., 2019).

### 2.6 Texture profile analysis (TPA)

The texture profile of the samples was determined with a Texture Analyzer^TM^ (Stable Micro System Ltd., Godalming, Surrey, GU7 1YL, UK). Cheese samples were molded in a dimension of 20 × 20 mm, and two‐bite compression experiments with a flat cylinder probe (25 mm diameter) were done at room temperature (±2°C). The strain rate at a speed of 1 mm/s and the maximum penetration rate of 10 mm were investigated. The textural parameters studied included hardness, cohesiveness, chewiness, resilience, and springiness (El‐Batawy & Soliman, 2017).

### 2.7 Total phenol content and antioxidant activity analysis

To determine the total phenol content (TPC) and antioxidant activity of cheeses, first, their methanol extracts were extracted. Five grams of sample was added to 5 ml of methanol and stirred for 5 min. Then, the extracts of cheeses were kept in the refrigerator for 2 h and centrifuged for 30 min at 8602 g (at 4°C). The final extracts were obtained after filtering the supernatant with Whatman paper No. 42 (Akan et al., 2021).

The TPC of cheeses was measured according to the Folin–Ciocalteu method. One milliliter of extract was added to 5 ml of diluted Folin–Ciocalteu reagent (1:10) and mixed for 6 min. Four milliliters of 20% sodium carbonate was then incorporated into the mixture. The mixture was kept at room temperature for 2 h, and its absorbance was recorded at 760 nm with a ultraviolet–visible (UV–vis) spectrophotometer. The TPC of samples was reported as mg gallic acid equivalents/g of cheese (mg GAE/g) (Pimentel‐González et al., 2015).

The antioxidant activity of the cheeses was measured by two methods, including 2,2‐diphenyl‐1‐picrylhydrazyl (DPPH) radical scavenging and 2,2′‐azino‐bis‐(3‐ethylbenzothiazoline‐6‐sulfonic acid) (ABTS) assays. To determine the DPPH radical scavenging activity, a methanol DPPH solution (0.2 mM DPPH in 80% methanol) was prepared. One hundred microliters of the sample was added to 100 μl of the DPPH solution and stirred. The resulting mixture was kept at room temperature for 1 h to complete the reaction. Finally, its absorbance (As) was read at 517 nm against control (methanol) (Ac). The DPPH radical scavenging activity of cheeses was obtained using the following equation and was expressed as a percentage (%) (Akan et al., 2021).

$$\text{DPPH radical scavenging activity}\;\left(\%\right)=\frac{\mathrm{Ac}-\mathrm{As}}{\mathrm{Ac}}\times 100$$

To determine the ABTS radical inhibitory activity of the cheeses, 10 ml of the ABTS solution (7 mM) was added to 10 ml of potassium persulfate (2.45 mM). The mixture was then stirred in the dark for 16 h. After that, 200 μl of the cheese extract was mixed with 2 ml of ABTS solution and kept for 6 min, and then its absorbance was recorded at 734 nm. The final results were reported in mg Trolox equivalent (TE) per g dw of cheese (Pérez‐Soto et al., 2021; Pimentel‐González et al., 2015).

### 2.8 Rheological properties

The small‐amplitude oscillatory shear measurements were performed by a Universal Dynamic Spectrometer, Paar Physica UDS 200, rheometer (Physica Messtechnik GmbH, Stuttgart, Germany). The primary viscoelastic terms (the storage, G′, and loss moduli, G″) along with phase angle tangent, tan (d), were determined. The measurement system consisted of two parallel plates with a diameter of 25 and a gap size of 1.0 mm (sample thickness). Samples were cut from the center of cheese blocks at 9 ± 1°C and were immediately placed in plastic bags, sealed, and equilibrated at room temperature (25 ± 1°C) for at least 5 h. A small piece of cheese was then placed on the lower plate and then the upper plate was slowly moved down until the preset gap size was reached. The extra cheese parts were trimmed off carefully with a razor blade and the sample was let relax for 15 min in the rheometer to allow stresses induced during sample handling to relax. The linear viscoelastic range was obtained by performing a strain sweep test at 0.1 Hz frequency as the percentage of strain values varied from 0.01 to 2.00%. A strain in the linear region was then selected (0.02) and a frequency sweep test was performed from 0.1 to 100 Hz. Means of two measurements for three replicates of cheese samples were reported (Karami et al., 2009).

### 2.9 Sensory evaluation

The sensory properties of cheese samples, including texture, color, taste, odor, and overall acceptance, were evaluated using a 5‐point Hedonic test (1: very bad, 2: bad, 3: medium, 4: good, 5: very good) by 10 panelists. After removing the cheese samples from the refrigerator and reaching the ambient temperature, the samples were coded and given to the panelists (Beigomi et al., 2013).

### 2.10 Statistical analysis

The mean values were statistically analyzed by SPSS software (version 22.0). The calculation was performed by one‐way analysis of variance (ANOVA) followed by Duncan multiply post hoc test at *p* < .05.

## 3 RESULTS AND DISCUSSION

### 3.1 Chemical composition

Changes in the chemical composition of the control ricotta cheese and samples containing free and nano‐encapsulated BSE during the 15‐day refrigerated storage period are presented in Table 1. The results showed that the incorporation of the free and nano‐encapsulated BSE to ricotta cheese formulation did not cause significant changes in moisture, protein, and fat contents of cheeses. The moisture, protein, and fat contents of the cheese samples were in the ranges of 51.77%–52.74%, 16.93%–17.54%, and 18.84%–19.82%, respectively. Moisture reduction was observed in all cheese samples during the 15‐day storage, which is due to the evaporation of moisture from the surfaces of the cheese (Pérez‐Soto et al., 2021). However, due to the hygroscopic characteristic of BSG, the presence of this gum in the nano‐capsules caused better moisture retention during storage. During the storage period, an increase in protein and fat content of the cheese samples was observed (*p* < .05). The increase in fat and protein content in the cheeses over time is probably due to the whey coming out of the curd (Kebary et al., 2018).

**TABLE 1:** Comparison of the chemical composition of the ricotta cheese samples during 15‐day refrigerated storage

Samples	Storage period (day)	Moisture content (%)	Protein content (%)	Fat content (%)
Control	Fresh	52.49 ± 0.25 ^ab^	16.93 ± 0.22 ^b^	18.90 ± 0.16 ^c^
	7	52.00 ± 0.21 ^cd^	17.19 ± 0.20 ^ab^	19.21 ± 0.07 ^b^
	15	51.77 ± 0.17 ^d^	17.53 ± 0.13 ^a^	19.76 ± 0.19 ^a^
3% Free BSE*	Fresh	52.56 ± 0.44 ^abc^	17.01 ± 0.29 ^b^	18.86 ± 0.22 ^c^
	7	52.35 ± 0.14 ^bc^	17.24 ± 0.17 ^ab^	19.28 ± 0.15 ^b^
	15	52.02 ± 0.19 ^cd^	17.51 ± 0.11 ^a^	19.79 ± 0.21 ^a^
5% Free BSE	Fresh	52.53 ± 0.39 ^abc^	17.04 ± 0.18 ^b^	18.88 ± 0.16 ^c^
	7	52.27 ± 0.26 ^bc^	17.30 ± 0.14 ^ab^	19.25 ± 0.13 ^b^
	15	51.98 ± 0.23 ^cd^	17.51 ± 0.13 ^a^	19.74 ± 0.13 ^a^
3% Nano BSE	Fresh	52.73 ± 0.17 ^a^	16.92 ± 0.25 ^b^	18.84 ± 0.21 ^c^
	7	52.63 ± 0.11 ^a^	17.20 ± 0.22 ^ab^	19.29 ± 0.11 ^b^
	15	52.47 ± 0.14 ^ab^	17.54 ± 0.16 ^a^	19.80 ± 0.15 ^a^
5% Nano BSE	Fresh	52.74 ± 0.20 ^a^	16.99 ± 0.18 ^b^	18.94 ± 0.14 ^c^
	7	52.72 ± 0.17 ^a^	17.22 ± 0.16 ^ab^	19.24 ± 0.12 ^b^
	15	52.54 ± 0.15 ^ab^	17.49 ± 0.17 ^a^	19.82 ± 0.14 ^a^

*Note*: Values represent mean (*n* = 3) ± SD. Different letters in each column represent significant difference at 5% level of probability among samples.

* BSE, Broccoli sprouts extract.

Similarly, El‐Den (2020) showed that incorporating curcumin bioactive compound to ricotta cheese had no significant effect on fat content, and the amounts of fat and protein of cheeses increased during storage. Farrag et al. (2020) stated that the control fresh cheese and cheeses with free and encapsulated olive polyphenols extract had the same fat amount ranging from 13.00% to 13.78%. Hamdy and Hafaz (2018) also found that adding rosemary, basil, and thyme to ricotta cheese had no significant effect on its chemical composition, however, during the 21‐day storage, no significant change was observed in the fat, moisture, and protein contents of cheeses. The fat and protein content reported by Pérez‐Soto et al. (2021) for controlling fresh cheese and cheeses containing *Opuntia oligacantha* microcapsule and nano‐emulsion were in the ranges of 22.43%–25.33% and 19.26%–22.65%, respectively, which were higher than the values obtained in the present study. However, they recorded lower moisture content for fresh cheeses (48.11–49.92%). The positive effect of gum on moisture retention in fresh cheeses was also observed by these researchers. Balabanova et al. (2020) found that the incorporation of the encapsulated pepper extracts to the Labneh cheese did not significantly change the fat, moisture, and protein contents of the cheeses, and the moisture content decreased and the fat and protein content increased during the storage period, but these changes were not statistically significant.

### 3.2 The pH and acidity

The pH and titratable acidity values of the ricotta cheeses containing different concentrations of free and nano‐encapsulated BSE during the 15‐day storage period at 4°C are compared in Table 2. As can be seen in the table, on the first day of the experiment, adding the free and encapsulated BSE significantly reduced the pH and increased the acidity of the ricotta cheeses (*p* < .05), however in this day, the type and concentration of BSE did not show a significant effect. Decreased pH and increased acidity due to the incorporation of free and nano‐encapsulated BSE are probably due to the presence of acidic compounds and phenolic acids in the extract (Hala et al., 2010). During the 15‐day storage, due to the partial decomposition of sugars and lactate production by microorganisms (Foda et al., 2009; Ramos et al., 2012), the pH of cheeses decreased and the acidity increased significantly (*p* < .05). The highest changes in these chemical parameters were related to the control sample, which is probably due to the higher growth of microorganisms in this sample. The pH and titratable acidity values of the cheese samples were in the ranges of 5.01–5.41 and 0.47–1.06%, respectively.

**TABLE 2:** Comparison of the pH and titratable acidity of the ricotta cheese samples during 15‐day refrigerated storage

Samples	Storage period (day)	pH	Titratable acidity (% LA)
Control	Fresh	5.41 ± 0.02 ^a^	0.47 ± 0.02 ^g^
	7	5.22 ± 0.00 ^f^	0.84 ± 0.05 ^bc^
	15	5.01 ± 0.02 ^h^	1.06 ± 0.06 ^a^
3% Free BSE*	Fresh	5.35 ± 0.01 ^b^	0.54 ± 004 ^f^
	7	5.25 ± 0.00 ^e^	0.71 ± 0.02 ^d^
	15	5.17 ± 0.02 ^g^	0.90 ± 0.05 ^b^
5% Free BSE	Fresh	5.33 ± 0.01 ^b^	0.55 ± 0.04 ^f^
	7	5.26 ± 0.01 ^de^	0.65 ± 0.04 ^de^
	15	5.19 ± 0.02 ^g^	0.83 ± 0.02 ^b^
3% Nano BSE	Fresh	5.33 ± 0.02 ^b^	0.57 ± 0.03 ^f^
	7	5.28 ± 0.01 ^d^	0.68 ± 0.03 ^de^
	15	5.20 ± 0.03 ^fg^	0.78 ± 0.02 ^c^
5% Nano BSE	Fresh	5.32 ± 0.02 ^bc^	0.55 ± 0.01 ^f^
	7	5.28 ± 0.02 ^cd^	0.65 ± 0.03 ^e^
	15	5.22 ± 0.03 ^efg^	0.77 ± 0.03 ^c^

*Note*: Values represent mean (*n* = 3) ± SD. Different letters in each column represent significant difference at 5% level of probability among samples.

* BSE: Broccoli sprouts extract.

A decrease in pH and increase in acidity amounts of ultrafiltered (UF) fresh cheeses with an increasing level of rosemary extract were also observed by Hala et al. (2010). Pérez‐Soto et al. (2021), Tripaldi et al. (2020), El‐Den (2020) also reported a decrease in the pH and increase in acidity values of fresh and ricotta cheeses during the storage period. Flores‐Mancha et al. (2021) demonstrated that the addition of beet extract‐loaded capsules to the yogurt had no significant effect on the pH and acidity, and during the storage, the pH values of samples decreased. Matseychik et al. (2019) reported that the acidity value of cottage cheese dessert containing rowanberry extract capsules was higher than that of the control. In the study conducted by Balabanova et al. (2020), the incorporation of encapsulated pepper extracts to the Labneh cheese did not show a significant effect on the pH and acidity of the samples, and the pH and acidity of cheeses decreased and increased during storage, respectively.

### 3.3 Textural analysis

Table 3 shows the texture parameters (hardness, cohesiveness, chewiness, springiness, and resilience) of the ricotta cheeses containing different concentrations of free and nano‐encapsulated BSE during the 15‐day storage period at 4°C. The chemical composition of cheeses is one of the important factors affecting their texture properties, so that if the wall material used to encapsulate bioactive compounds can increase the total solids and protein content of the cheeses, it can also increase the hardness of the product (Farrag et al., 2020). Since the BSE‐loaded nano‐capsules produced in this study did not show a significant effect on the total protein and fat of ricotta cheese at the beginning of the cold storage period, they did not show any effect on the textural parameters on this day. Over time, the hardness values of the cheeses showed a significant increase, and the chewiness values of the samples also gradually increased (*p* < .05), which is probably due to the decrease in moisture content of cheese samples. Since the cheeses containing nano‐encapsulated BSE had better moisture retention ability than other samples, the hardness and chewiness values of these samples were less than the others. During the cold storage period, no significant change was observed in the cohesiveness, springiness, and resilience of the cheeses. The hardness, cohesiveness, chewiness, springiness, and resilience values of the cheese samples were in the ranges of 95.40–150.51 g, 0.25–0.32, 92.32–95.43 g/mm, 6.990–6.995 mm, and 0.07–0.08 mm, respectively.

**TABLE 3:** Comparison of the textural parameters of the ricotta cheese samples during 15‐day refrigerated storage

Samples	Storage period (day)	Hardness (g)	Cohesiveness	Chewiness (g/mm)	Springiness (mm)	Resilience (mm)
Control	Fresh	96.52 ± 1.12 ^d^	0.27 ± 0.06 ^a^	93.34 ± 0.82 ^bc^	6.995 ± 0.005 ^a^	0.07 ± 0.00 ^a^
	7	129.73 ± 1.20 ^b^	0.30 ± 0.03 ^a^	94.42 ± 0.79 ^ab^	6.994 ± 0.003 ^a^	0.08 ± 0.01 ^a^
	15	150.51 ± 1.94 ^a^	0.29 ± 0.03 ^a^	95.43 ± 0.57 ^a^	6.991 ± 0.002 ^a^	0.07 ± 0.00 ^a^
3% Free BSE*	Fresh	96.31 ± 1.36 ^d^	0.25 ± 0.06 ^a^	92.92 ± 0.65 ^c^	6.994 ± 0.004 ^a^	0.07 ± 0.01 ^a^
	7	128.17 ± 1.28 ^b^	0.28 ± 0.02 ^a^	94.26 ± 0.67 ^ab^	6.992 ± 0.002 ^a^	0.07 ± 0.00 ^a^
	15	148.23 ± 1.01 ^a^	0.32 ± 0.05 ^a^	94.39 ± 0.75 ^ab^	6.993 ± 0.007 ^a^	0.08 ± 0.01 ^a^
5% Free BSE	Fresh	97.74 ± 1.26 ^d^	0.27 ± 0.02 ^a^	93.10 ± 0.47 ^c^	6.994 ± 0.004 ^a^	0.07 ± 0.01 ^a^
	7	128.04 ± 0.98 ^b^	0.28 ± 0.02 ^a^	93.55 ± 0.58 ^bc^	6.993 ± 0.001 ^a^	0.08 ± 0.01 ^a^
	15	147.04 ± 1.52 ^a^	0.30 ± 0.04 ^a^	94.22 ± 0.52 ^ab^	6.993 ± 0.002 ^a^	0.07 ± 0.00 ^a^
3% Nano BSE	Fresh	95.62 ± 1.17 ^d^	0.30 ± 0.03 ^a^	92.32 ± 0.70 ^c^	6.994 ± 0.002 ^a^	0.07 ± 0.00 ^a^
	7	119.78 ± 1.40 ^c^	0.31 ± 0.04 ^a^	92.64 ± 0.49 ^c^	6.993 ± 0.002 ^a^	0.08 ± 0.01 ^a^
	15	129.52 ± 0.83 ^b^	0.31 ± 0.03 ^a^	93.32 ± 0.59 ^bc^	6.993 ± 0.004 ^a^	0.08 ± 0.01 ^a^
5% Nano BSE	Fresh	95.40 ± 1.45 ^d^	0.31 ± 0.05 ^a^	92.74 ± 0.79 ^c^	6.994 ± 0.003 ^a^	0.07 ± 0.01 ^a^
	7	119.17 ± 0.89 ^c^	0.29 ± 0.02 ^a^	93.37 ± 0.35 ^bc^	6.990 ± 0.007 ^a^	0.07 ± 0.00 ^a^
	15	127.24 ± 2.46 ^b^	0.27 ± 0.04 ^a^	93.25 ± 0.62 ^bc^	6.994 ± 0.002 ^a^	0.07 ± 0.00 ^a^

*Note*: Values represent mean (*n* = 3) ± SD. Different letters in each column represent significant difference at 5% level of probability among samples.

* BSE: Broccoli sprouts extract.

Shamsia and El‐Ghannam (2017), and El‐Batawy and Soliman (2017) also reported a significant increase in the hardness of ricotta cheese during the storage period. Pérez‐Soto et al. (2021) found that the addition of *Opuntia oligacantha* microcapsule and nano‐emulsion to fresh cheese reduced the hardness and chewiness of cheeses compared to control, but did not have an effect on elasticity and cohesiveness parameters. During 45‐day storage, the hardness of the cheeses decreased but no significant change was observed in other textural parameters. Fuentes et al. (2015) also observed no significant change in the cohesiveness of fresh cheese during the 24‐day storage. Artiga‐Artigas et al. (2017) reported a decrease in the hardness of cheeses containing oregano essential oil nano‐emulsion. Farrag et al. (2020) showed that in the control fresh cheese and cheeses containing free and encapsulated olive polyphenols extract during the 30‐day storage period, there was no significant change in the textural parameters of cheeses, including hardness, springiness, cohesiveness, gumminess, and chewiness.

### 3.4 Total phenol content (TPC) and antioxidant activity

**FIGURE 1:**
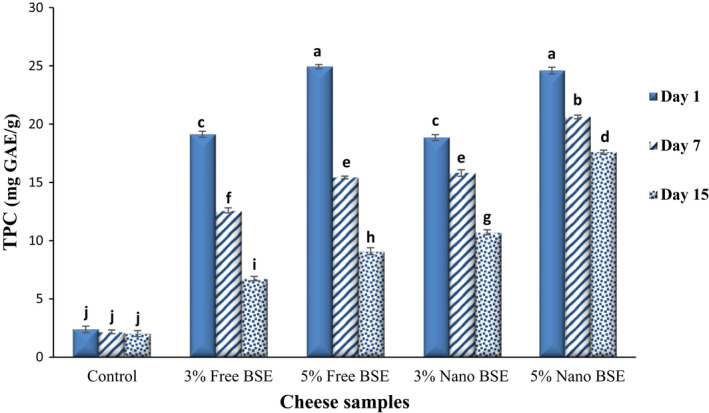
Comparing the total phenol content (TPC) (mg gallic acid equivalent (mg GAE)/g of cheese) of the ricotta cheese samples during 15‐day refrigerated storage period.

Phenolic compounds are the most important metabolites and bioactive compounds in various plant sources that often show remarkable and significant functional activates such as antioxidant, anti‐inflammatory, and antimicrobial activities. The TPC of the ricotta cheeses containing different concentrations of free and nano‐encapsulated BSE during the 15‐day storage period at 4°C is compared in Figure 1.

The results demonstrated that the addition of the free and nano‐encapsulated BSE to the ricotta cheeses caused a significant increase in TPC of samples, and with increasing the concentration of extracts from 3% to 5%, a significant increase in TPC was observed (*p* < .05). However, on the first day of the experiment, there were no significant differences between the samples containing free and encapsulated BSE. Over time, the TPC of the samples decreased (*p* < .05). The TPC of the cheeses was in the range of 2.39–24.94 mg GAE/g of cheese on the first day and reached 2.03–17.59 mg GAE/g of cheese on the last day. The highest intensity to decrease was related to the cheeses containing the free extract. Decreased TPC of the cheeses containing free BSE during the storage period is probably due to the sensitivity of free phenolic compounds to oxidation and degradation over time (Mohamed et al., 2018). The nano‐encapsulation process of the extract resulted in better maintenance of phenolic compounds and their controlled release. Chlorogenic acid, *p*‐coumaric acid, ferulic acid, gentistic acid, sinapic acid, robinin, and so on, are important phenolic compounds in the broccoli sprouts (Paśko et al., 2018).

**FIGURE 2:**
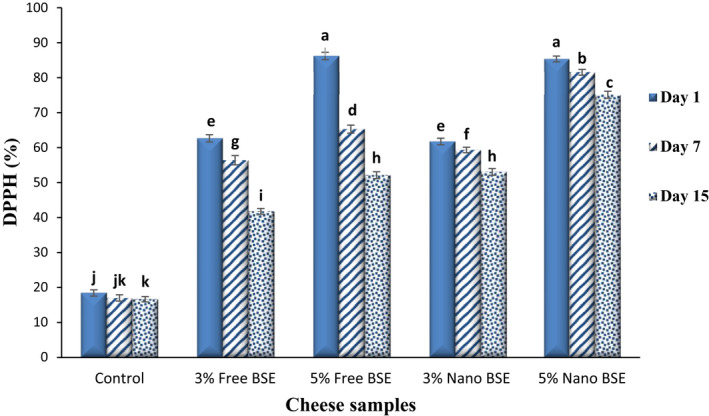
Comparing the 2,2‐diphenyl‐1‐picrylhydrazyl (DPPH) (%) of the ricotta cheese samples during 15‐day refrigerated storage period.

**FIGURE 3:**
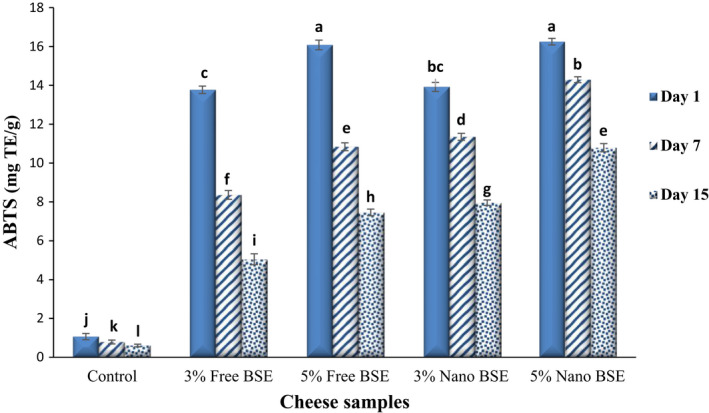
Comparing the 2,2′‐azino‐bis‐(3‐ethylbenzothiazoline‐6‐sulfonic acid) (ABTS (mg trolox equivalent (mg TE)/g of cheese) of the ricotta cheese samples during 15‐day refrigerated storage period.

The antioxidant activity of the ricotta cheese samples was measured by two methods of DPPH and ABTS, and their results are given in Figures 2 and 3, respectively. As expected, by adding the free and nano‐encapsulated BSE to the cheese formulation, the antioxidant activity of the cheeses significantly increased (*p* < .05), and a positive and direct relationship was observed between the concentration of the extract and the antioxidant activity, so that increasing the concentration of BSE from 3% to 5% showed a significant increase in antioxidant activity (*p* < .05).

Due to the reduction of the TPC of the cheeses during the 15‐day storage period, their antioxidant activity (DPPH and ABTS) also decreased significantly (*p* < .05). Since in the cheese samples containing free BSE, the highest rate of loss of bioactive and phenolic compounds occurred, the antioxidant activity of these samples also decreased over time compared to other samples. The DPPH and ABTS values of the ricotta cheese samples on the first day were in the ranges of 18.44–86.21% and 1.07–16.25 mg TE/g of cheese, respectively, and on the last day, they reached 16.66–75.13% and 0.61–10.79 mg TE/g of cheese, respectively. The antioxidant activity of the BSE is due to the presence of bioactive and functional compounds, especially phenolic compounds, flavonoids (Paśko et al., 2018), and sulforaphane (Akbari & Namazian, 2020), in it. Previous studies have confirmed the antioxidant activity of BSE (Aloo et al., 2021; Pająk et al., 2014). The ABTS radicals are soluble in organic solvents and water and can determine the antioxidant capacity of both hydrophobic and hydrophilic compounds (Shah & Modi, 2015). The DPPH free radical is a stable free radical that is widely used to study the activities of antioxidants (Mohammad Kheshtchin et al., 2022). The DPPH radical scavenging assay is the most sensitive and simple spectrophotometric method for antioxidant capacity of plant extract determination (Farahmandfar et al., 2019). Given the good results obtained by the three main tests in our previous study (Azarashkan, Farahani, et al., 2022) and since the ABTS and ferric reducing antioxidant power (FRAP) tests are complementary tests, as well as more common applications of DPPH and ABTS to determine antioxidant properties, these methods are used in this study.

Increased phenol content and antioxidant activity of UF‐soft cheeses were observed by adding rosemary extract and increasing its concentration by Hala et al. (2010). Hamdy and Hafaz (2018) demonstrated that the ricotta cheeses containing rosemary, basil, and thyme had higher phenol content and antioxidant activity than the control cheese and during the storage period in all samples the number of phenols and antioxidant activity decreased. Decreases in phenolic compounds and antioxidant activity of cheeses during storage have also been reported by some researchers (El‐Den, 2020; Pasini Deolindo et al., 2019). Pérez‐Soto et al. (2021) also reported a significant increase in the phenol content and antioxidant activity of fresh cheese due to the incorporation of *Opuntia oligacantha* microcapsule and nano‐emulsion to cheeses. Matseychik et al. (2019) also found that adding rowanberry extract capsules to cottage cheese dessert significantly increased antioxidant activity. Farrag et al. (2020) demonstrated that in white fresh cheeses containing free olive polyphenols extract during the 30‐day storage period, the phenol content and antioxidant activity significantly decreased, but in cheeses containing encapsulated extract, no significant change in these functional parameters was observed over time. A decrease in the antioxidant activity of cottage cheeses containing free mushroom extracts and the significant increase in the antioxidant activity of cheeses containing encapsulated mushroom extracts during the storage period were also observed (Ribeiro et al., 2015).

### 3.5 Rheological properties

**FIGURE 4:**
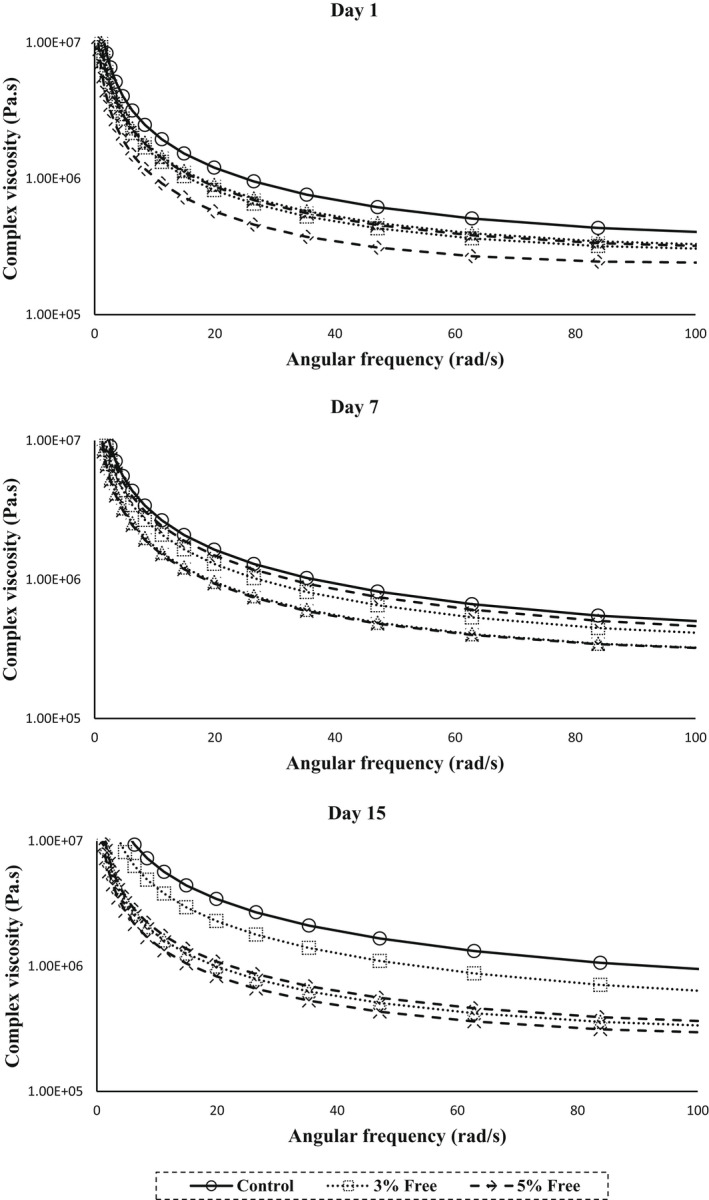
Changes in the complex viscosity (Pa.s) of ricotta cheeses versus angular frequency on different days of refrigeration.

Changes in the complex viscosity (*ƞ) values of different samples of ricotta cheese versus angular frequency on different storage days are shown in Figure 4.

As can be seen in the figure, in all studied days, with increasing frequency, the complex viscosity decreased nonlogarithmically, which indicates the thixotropic behavior of the cheese produced. Since complex viscosity is a measure of total hardness, the addition of BSE‐loaded nano‐capsules to ricotta cheese formulation reduced the amount of complex viscosity. The presence of BSG in the coating of nano‐capsules due to moisture retention in cheese samples caused a decrease in the complex viscosity. Hesarinejad et al. (2021) also reported a decrease in the complex viscosity of ricotta cheese with increasing the angular frequency. Decreased complex viscosity of yogurt samples due to the use of hydrocolloids has also been observed by Zamani et al. (2015).

**FIGURE 5:**
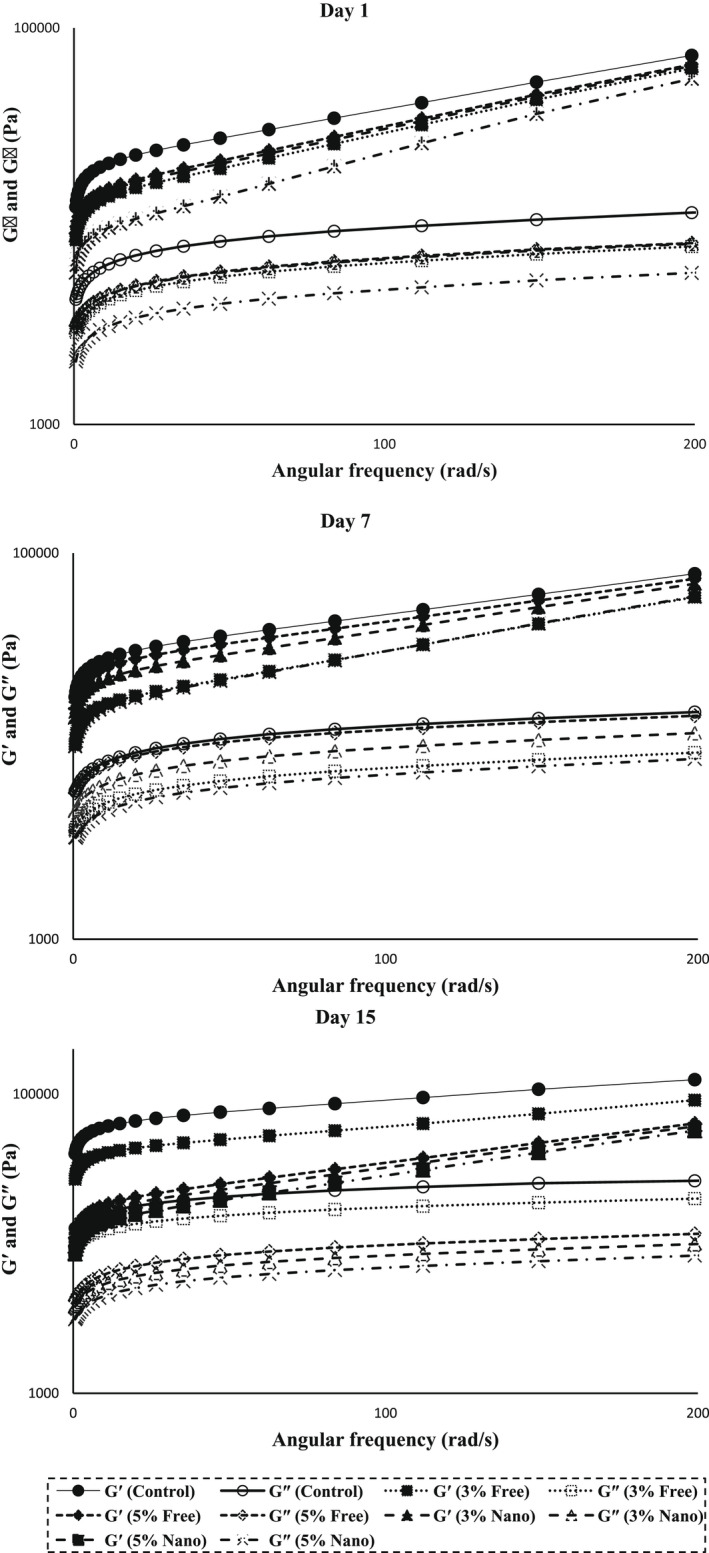
Changes in the G′ and G″ (Pa) of ricotta cheeses versus angular frequency on different days of refrigeration.

The storage modulus or shear modulus (G′) indicates the amount of elastic behavior and the amount of energy recovered per unit volume per strain wave cycle, and the loss modulus or viscose modulus (G″) indicates the amount of flow behavior and the amount of energy wasted per unit volume per wave cycle of strain. In general, the storage and loss modulus represent the elastic and viscose characteristics of food products, respectively. These two parameters are related to the softness of the cheese texture, and the higher their value for the cheese sample, the more firm and undesirable the texture (Madadlou et al., 2005). Changes in the storage and loss modulus of ricotta cheeses versus angular frequency on different days of refrigeration are presented in Figure 5 and show that both G′ and G″ were frequency‐dependent and showed a similar trend, so that in all cheese samples and on all days studied in this research, the G′ and G″ values increased with increasing frequency.

This behavior is called a rheologically weak gel. At all frequencies, the G′ was noticeably higher than the G″, which indicates the dominant role of the elasticity in the viscosity, or elastic nature of the cheeses. Thus, the cheese samples showed solid behavior. The solid behavior of cheeses containing BSE‐loaded nano‐capsules was lower than those of the control samples and cheese containing free BSE, which is related to the retention of moisture by BSG. Generally, there is an inverse relationship between moisture content and the G′ and G″ of cheeses, so that the reduction of moisture in cheese samples causes their texture to be firmer and more solid, and therefore increases the G′ and G″. Hesarinejad et al. (2021) similarly found that the ricotta cheeses exhibited viscoelastic behavior (shear‐thinning) and that the amount of G′ in this cheese was higher than the G″. These researchers attributed this to the internal relationship between the gel‐like network and the elastic behavior. Karami et al. (2009) also reported that the G′ of Feta cheese was higher than the G″. Similarly, in their research, the G′ and G″ increased over time. Rubel et al. (2019) also observed that with increasing frequency, the G′ and G″ of cheeses increased and the G′ was higher than the G″ at all frequencies.

### 3.6 Sensory evaluation

Sensory properties of the ricotta cheeses containing different concentrations of free and nano‐encapsulated BSE, including texture, taste, color, odor, and overall acceptability, were evaluated during the 15‐day storage period at 4°C, and the results are shown in Table 4. Incorporation of free and nano‐encapsulated BSE had no significant effect on the texture, color, and odor of cheeses on the first day, but with the addition of free BSE, taste and overall acceptability scores significantly decreased (*p* < .05). The nano‐encapsulation process covered the taste of BSE. During the storage period, due to the chemical and oxidative reactions and microbial growth, the sensory scores of the cheese samples decreased (*p* < .05), and the highest decrease in sensory scores was observed in the control sample, followed by the sample containing 3% free BSE. The addition of BSE, especially its high level, reduced the rate of destructive reactions in the cheeses and maintained the sensory quality of the samples during the refrigerated storage period. Due to the maintenance of bioactive and functional compounds in cheeses containing nano‐encapsulated BSE, the sensory quality of these samples was good until the last day of cold storage.

**TABLE 4:** Comparison of the sensory properties of the ricotta cheese samples during 15‐day refrigerated storage

Samples	Storage period (day)	Texture	Flavor	Color	Odor	Overall acceptability
Control	Fresh	4.80 ± 0.22 ^a^	4.90 ± 0.16 ^a^	4.80 ± 0.22 ^a^	4.80 ± 0.22 ^a^	4.90 ± 0.16 ^a^
	7	3.00 ± 0.00 ^e^	3.20 ± 0.22 ^d^	3.00 ± 0.00 ^d^	2.90 ± 0.16 ^d^	3.00 ± 0.00 ^e^
	15	2.50 ± 0.26 ^f^	2.80 ± 0.22 ^d^	2.70 ± 0.24 ^e^	2.40 ± 0.26 ^e^	2.50 ± 0.26 ^f^
3% Free BSE*	Fresh	4.90 ± 0.16 ^a^	3.80 ± 0.22 ^c^	4.80 ± 0.22 ^a^	4.60 ± 0.26 ^ab^	4.00 ± 0.00 ^c^
	7	3.60 ± 0.26 ^d^	3.80 ± 0.22 ^c^	4.00 ± 0.00 ^c^	4.00 ± 0.00 ^c^	3.50 ± 0.26 ^d^
	15	3.10 ± 0.16 ^e^	3.10 ± 0.16 ^d^	3.20 ± 0.24 ^d^	3.20 ± 0.22 ^d^	3.00 ± 0.00 ^e^
5% Free BSE	Fresh	4.80 ± 0.22 ^a^	3.00 ± 0.00 ^d^	4.40 ± 0.26 ^ab^	4.70 ± 0.28 ^ab^	4.00 ± 0.00 ^c^
	7	4.50 ± 0.26 ^ab^	3.00 ± 0.00 ^d^	4.40 ± 0.26 ^ab^	4.00 ± 0.00 ^c^	4.00 ± 0.00 ^c^
	15	4.00 ± 0.00 ^c^	3.00 ± 0.00 ^d^	3.80 ± 0.22 ^c^	3.80 ± 0.22 ^c^	3.50 ± 0.26 ^d^
3% Nano BSE	Fresh	4.90 ± 0.16 ^a^	4.90 ± 0.16 ^a^	4.80 ± 0.22 ^a^	4.80 ± 0.22 ^a^	4.90 ± 0.16 ^a^
	7	4.50 ± 0.26 ^ab^	4.30 ± 0.24 ^b^	4.50 ± 0.26 ^ab^	4.60 ± 0.26 ^ab^	4.50 ± 0.26 ^ab^
	15	4.00 ± 0.00 ^c^	4.00 ± 0.00 ^c^	4.30 ± 0.24 ^b^	4.30 ± 0.24 ^b^	4.30 ± 0.24 ^b^
5% Nano BSE	Fresh	4.90 ± 0.16 ^a^	4.80 ± 0.22 ^a^	4.80 ± 0.16 ^a^	4.80 ± 0.22 ^a^	4.90 ± 0.16 ^a^
	7	4.70 ± 0.24 ^ab^	4.80 ± 0.22 ^a^	4.50 ± 0.26 ^ab^	4.50 ± 0.26 ^ab^	4.60 ± 0.26 ^ab^
	15	4.30 ± 0.24 ^b^	4.30 ± 0.24 ^b^	4.20 ± 0.22 ^bc^	4.30 ± 0.24 ^b^	4.30 ± 0.24 ^b^

*Note*: Values represent mean (*n* = 3) ± SD. Different letters in each column represent significant difference at 5% level of probability among samples.

* BSE: Broccoli sprouts extract.

Hala et al. (2010) stated that adding rosemary extract did not adversely affect the sensory properties (appearance, flavor, texture, and overall acceptability) of soft cheese. Hamdy and Hafaz (2018) showed that the addition of rosemary, basil, and thyme had a positive effect on the sensory characteristics of ricotta cheese, and a slight decrease in the sensory acceptance of the cheeses was observed during the 21‐day storage period. El‐Den (2020) found that the incorporation of curcumin to ricotta cheese maintained the sensory quality of the cheeses during storage. In the research conducted by Farrag et al. (2020), the addition of olive polyphenols extract caused a slight and nonsignificant reduction in the sensory acceptance of the white soft cheeses, but the incorporation of encapsulated olive polyphenols extract significantly increased the overall acceptance of cheeses. Adding the encapsulated pepper extracts to the Labneh cheese also improved the sensory acceptance of the samples during the storage period (Balabanova et al., 2020).

## 4 CONCLUSION

The results of this study demonstrated that the use of the nano‐encapsulated BSE in ricotta cheese resulted in better moisture retention and reduced the increased rate of the hardness of the cheeses texture over time. The nano‐encapsulation of BSE with nano‐liposome and BSG was able to improve the stability of phenolic and bioactive compounds of BSE and in this way, maintain the functional compounds in cheeses and improve antioxidant activity of samples during the storage period. Therefore, according to the results obtained in this study, the nano‐encapsulated BSE can be effectively used to produce functional ricotta cheese with desirable and good antioxidant activity.

## CONFLICTS OF INTEREST

The authors declare that they have no conflicts of interest to disclose.

## Data Availability

Data Availability The data that support the findings of this study are available from the author, Zahra Azarashkan (Azarashkan.za@gmail.com), upon reasonable request.
